# Lipid Membrane Topographies Are Regulators for the Spatial Distribution of Liquid Protein Condensates

**DOI:** 10.1021/acs.nanolett.3c04169

**Published:** 2024-04-05

**Authors:** Chae Yeon Kang, Yoohyun Chang, Katja Zieske

**Affiliations:** Biophysics, Max Planck Institute for the Science of Light, 91058 Erlangen, Germany

**Keywords:** Lipid membrane, biomolecular condensates, microstructures, soft matter

## Abstract

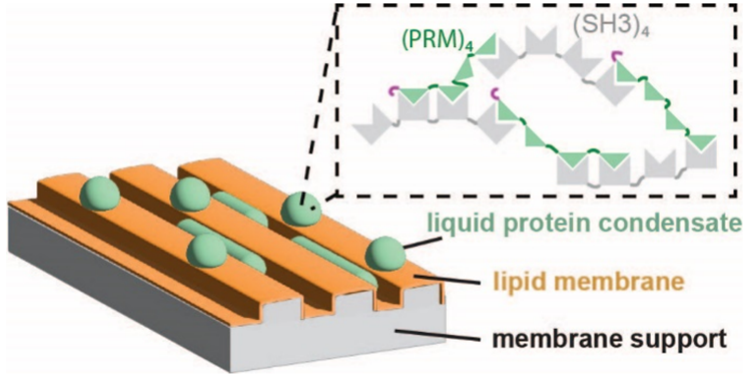

Liquid protein condensates play important roles in orchestrating subcellular organization and as biochemical reaction hubs. Recent studies have linked lipid membranes to proteins capable of forming liquid condensates, and shown that biophysical parameters, like protein enrichment and restricted diffusion at membranes, regulate condensate formation and size. However, the impact of membrane topography on liquid condensates remains poorly understood. Here, we devised a cell-free system to reconstitute liquid condensates on lipid membranes with microstructured topographies and demonstrated that lipid membrane topography is a significant biophysical regulator. Using membrane surfaces designed with microwells, we observed ordered condensate patterns. Furthermore, we demonstrate that membrane topographies influence the shape of liquid condensates. Finally, we show that capillary forces, mediated by membrane topographies, lead to the directed fusion of liquid condensates. Our results demonstrate that membrane topography is a potent biophysical regulator for the localization and shape of mesoscale liquid protein condensates.

The precise spatial and temporal organization of cellular components plays a fundamental role in numerous life processes, including cell division, cellular migration, and cellular polarization. Traditionally, the plasma membrane and membrane-bound organelles have been considered central hubs of spatial cellular organization. However, in the past decade, a paradigm shift has emerged in our understanding of cellular organization with the investigation of membrane-less organelles, commonly referred to as “liquid protein condensates” or “biomolecular condensates”.^1−7^ Liquid protein condensates are formed through the assembly of proteins or nucleic acids through unstructured domains or weak multivalent interactions.^8,9^

Both liquid condensates and lipid membranes serve as orchestrators of intracellular spatial organization, and their physical properties and biochemical functions have been the subject of intensive investigation.^10,11^ Emerging evidence shows an interplay between lipid membranes and liquid protein condensates.^12,13^ For instance, membrane components, such as transmembrane receptors, that are involved in cellular signaling processes are often organized in nano- to micrometer-scale clusters.^12^ Despite recent progress in understanding these interactions, questions remain regarding the systems-level consequences arising from the interaction between liquid protein condensates and biological interfaces. Specifically, the question of how geometric features of lipid membranes affect liquid protein condensates is still understudied.^14^ Recent studies describe the remodeling of lipid membranes by liquid protein condensates. Examples of such findings include the observation of liquid protein condensates remodeling plant vacuolar membranes^15^ and the remodeling of membranes by endocytic protein condensates.^16^ In addition, previous studies demonstrate a role of lipid membranes in modulating the concentration threshold for condensate formation and controlling the size of liquid protein condensates. This modulation occurs through the enrichment of condensate components and by limiting diffusion.^17^

Biomolecular wetting phenomena are emerging as an additional framework for the organization of liquid protein condensates at biological interfaces.^14^ Notable examples include its regulation of autophagy,^18^ its role in forming domains,^19^ and its association with various diseases.^20−24^ Thus, the hypothesis arises that membrane topography and associated capillary forces may be regulatory factors in the organization of liquid protein condensates. However, a major factor contributing to the limited exploration of how membrane shape influences condensate assembly and distribution is the inherent complexity of living cells. The large number of molecular interactions that occur within cells and the complexity of cellular shapes represent a challenge in systematically unraveling the role of membrane topography for liquid protein condensate formation and behavior in living cells.

Cell-free systems are intriguing tools that offer the advantage of disentangling the complexity of living cells, allowing a systems-level exploration of the biophysical mechanisms underlying molecular organization. Consequently, the cell-free reconstitution of biological components in precisely controlled environments is a promising strategy for the systematic investigation of protein interactions with lipid membranes. Recent cell-free studies probed the interaction of liquid protein condensates with supported lipid membranes^9^ and giant unilamellar vesicles^25,26^ and revealed, for instance, that liquid protein condensates on supported lipid membranes have the capacity to promote local assembly of cytoskeletal structures.^27,28^ Other cell-free experiments demonstrate the role of liquid protein condensates in bending and remodeling lipid membranes.^29,30^ These experiments on spherical vesicles established wetting as an efficient mechanism for membrane deformation. However, spherical and flat membranes, due to their uniform topography, may not fully capture the nuanced effects of cellular topography. A controlled assay for the systematic study of liquid condensate assembly in the context of various lipid membrane topographies is required to study condensates in the context of membranes mimicking cellular topographies.

Here, we explored the intricate assembly dynamics of liquid condensates on topographically structured membranes in a well-controlled environment. We developed a cell-free system composed of topographically structured, supported lipid membranes, and membrane interacting liquid protein condensates, and demonstrate three main findings. First, we show that liquid protein condensates preferentially localize at the periphery of microstructured membrane compartments. This preference underscores the role of membrane topography in governing the assembly of liquid protein condensates through capillary forces. Second, we demonstrate that liquid condensates deform within the confines of membrane-clad microgrooves. These observations demonstrate the regulation of condensate shape by membrane topography. Finally, our experiments reveal the presence of directionally defined forces acting upon liquid protein condensates in the context of topographically structured membranes. We demonstrate that liquid protein condensates move toward specific locations, defined by the specific arrangements of membrane topography and liquid condensates. These orchestrated interactions result in the emergence of spatial condensate patterns, offering insight into the underlying mechanisms for generating intracellular order.

## Reconstituting Liquid Protein Condensates in Membrane-Clad Microcompartments

**Figure 1:**
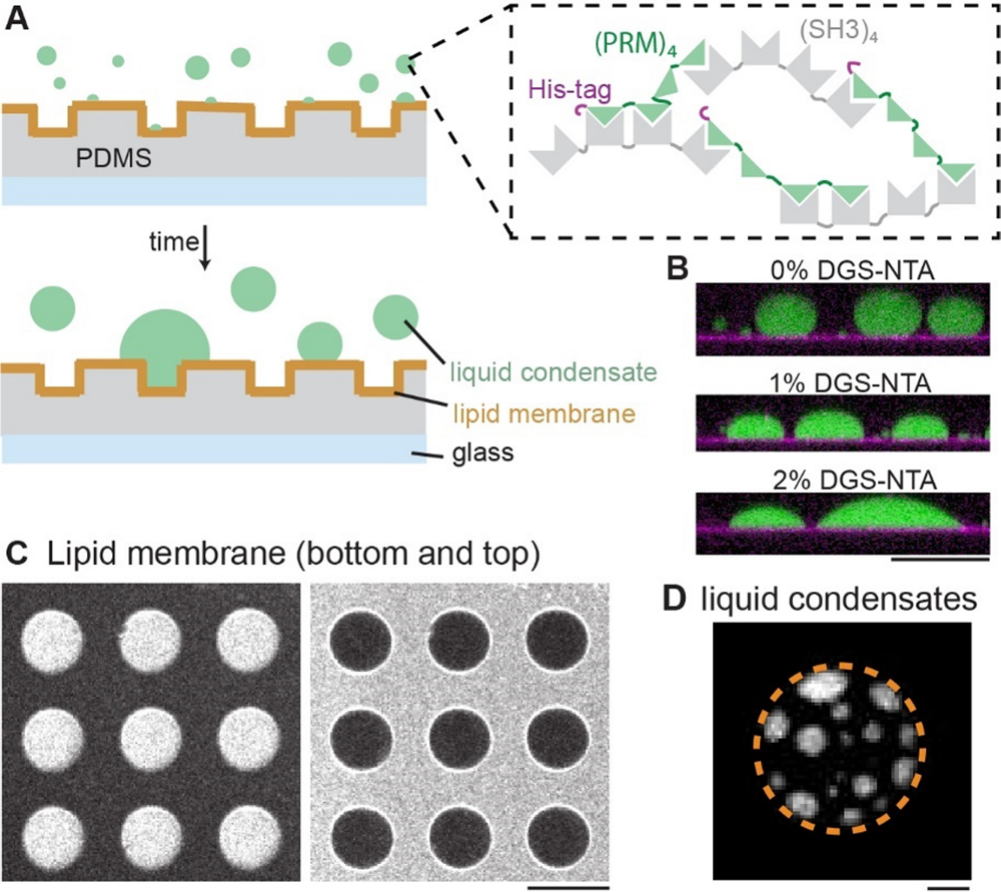
A cell-free assay was established for the spatiotemporal characterization of liquid protein condensates on topographically structured membranes. (A) Schematic of the experiment. Microstructured PDMS surfaces were clad with supported lipid membranes. The lipid membranes are supplemented with 2% DGS-NTA to mediate the interaction of his-tagged proteins with the membrane. When the proteins PRM_4_ and SH3_4_ are mixed, they form liquid protein condensates. The liquid protein condensates bind to the membrane through the histidine-tag of PRM_4_. (B) Side view of liquid protein condensates (green) on supported lipid membranes with 0%, 1% and 2% DGS-NTA respectively. Lipid membranes were labeled with 0.05% DiI (magenta). Protein concentrations: 50 μM PRM_4_, 1% Alexa488-labeled PRM_4_ and 50 μM SH3_4_. Scale bar: 50 μm (C) Confocal image of a lipid membrane doped with 0.05% DiI, illustrating that lipid membranes are cladding the bottom and top of PDMS microstructures. Scale bar: 50 μm (D) When 50 μM PRM_4_, 1% Alexa488-labeled PRM_4_ and 50 μM SH3_4_ were added to microstructured membranes, condensates (white) started to form and attached to the lipid membranes (2% DGS-NTA) through the histidine-tag of PRM_4_. The boundary of the microcompartments are indicated by the dotted orange line. Diameter of microcompartment: 40 μm. Depth of microstructures: 7 μm. Scale bar: 10 μm.

We were intrigued by the question of how variations in cell shape may influence processes associated with liquid condensates. However, despite the demonstration of liquid protein condensates occurring at cellular membranes and suggestions that capillary forces due to membrane topography play a role in their organization,^14^ an experimental system for the systematic investigation of liquid protein condensates in the context of topographically structured membranes was still missing. To address this gap in studying the organization of liquid protein condensates, we developed a cell-free approach designed to reconstitute liquid protein condensates on supported lipid membranes displaying defined topographical structures. Employing photolithography and soft molding techniques, we engineered a thin polydimethylsiloxane (PDMS) layer on top of a glass coverslip,^31^ featuring cylindrical microcompartments. Subsequently, we clad these PDMS microstructures with lipid membranes made of the lipid components DOPC and DGS-NTA. Thereby, DGS-NTA served as an engineering solution for tethering liquid protein condensates to the lipid membrane (Figure 1A).

For the formation of liquid protein condensates, we purified two previously described synthetic proteins,^32^ each comprising four motif repeats. The first, (SH3)_4_ consisted of four SRC homology 3 (SH3) domains, while the second protein, PRM_4_, was composed of four proline-rich motif (PRM) repeats, representing target sequences of SH3 domains. These proteins have previously been demonstrated to form liquid condensates above a critical concentration, and this two-component protein system offers a high degree of control over phase separation behavior through the modulation of protein concentrations and valency.^9^

We characterized the impact of DGS-NTA containing membranes on liquid protein condensates on flat supported membranes. With concentrations of (SH3)_4_ and PRM_4_ above the critical concentration for liquid protein condensate formation, small condensates formed in the buffer solution and settled on the membrane due to gravity. As time progressed these condensates grew through interaction with additional proteins and fusion with other condensates. On membranes lacking DGS-NTA, condensates remained nearly spherical with a large surface contact angle. The presence of DGS-NTA induced wetting of membranes by liquid protein condensates and the surface contact angle decreased with increasing amounts of DGS-NTA (Figure 1B). These findings align with wetting phenomena previously observed on giant unilamellar vesicles, were wetting was induced by modulating membrane charge or the ionic strength of the solution.^26^ Further, we added protein condensate components to DGS-NTA containing membranes with microtopographies and verified the emergence of liquid protein condensates with characteristic droplet-like appearance and wetting-characteristics (Figure 1C,D). Our results highlight the role of membrane linkers as mediators for wetting phenomena. Given the challenges of altering intracellular ionic strength or lipid composition in living cells, our results suggest membrane linkage as an efficient mediator for wetting phenomena.

## Assembly of Liquid Protein Condensates within Cylindrical Microcompartments and Microgrooves

**Figure 2:**
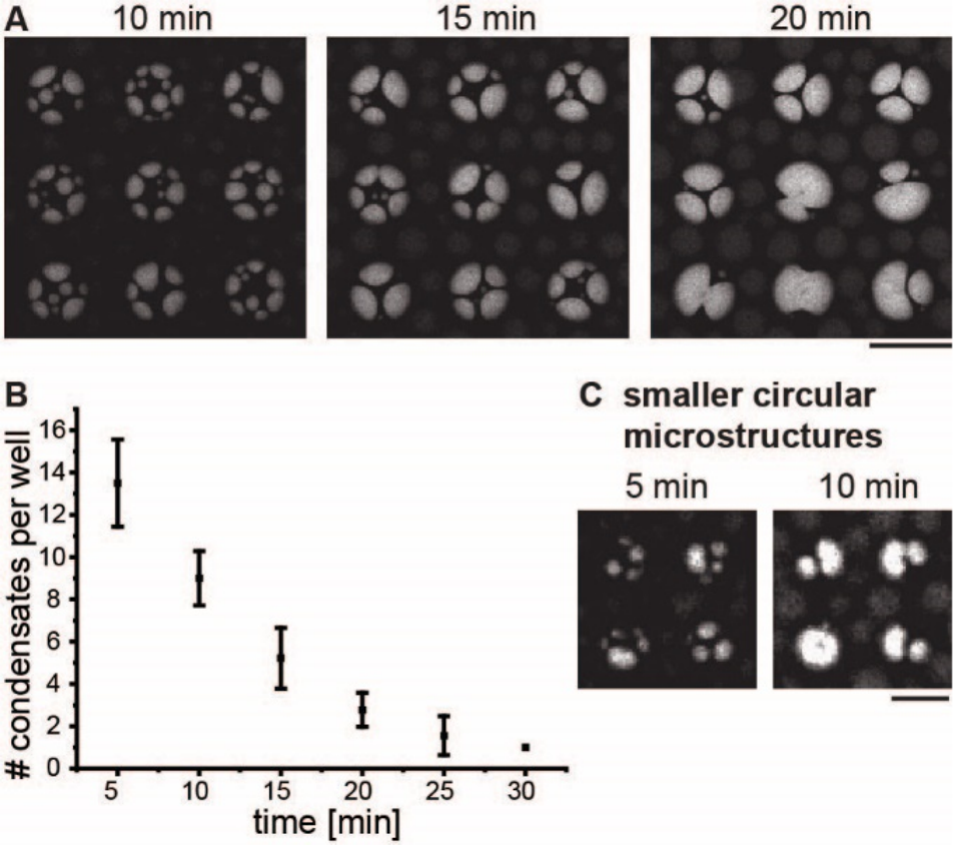
Peripheral assembly of liquid protein condensates within lipid membrane cavities. (A) Confocal time-lapse images illustrate the spatial distribution of fluorescently labeled liquid protein condensates within round micro cavities over time. The liquid protein condensates fuse to generate larger condensates and preferentially localize to the edges of the membrane cavities. Diameter of microcavity: 40 μm. Scale bar: 50 μm (B) The number of liquid protein condensates decreases with time until the whole cavity is filled with one large condensate. *n* > 10, error bar: standard deviation. (C) Membrane cavities with a smaller diameter of 20 μm are filled earlier than the larger membrane cavities. Depth of microstructures: 7 μm. Scale bar: 20 μm.

Wettability and capillary actions are related phenomena acting at the fluid–condensate–membrane interfaces to minimize the surface energy of these interfaces. To explore whether capillary actions affect the distribution of liquid protein condensates we investigated the influence of condensate size on their spatial distribution within cylindrical compartments acquiring time-lapse confocal microscopy images. As liquid condensates exhibit fusion and growth over time, we anticipated the observation of larger condensates over time. Indeed, as time progressed, we observed a notable increase in condensate volume, together with a reduction in the number of liquid protein condensates in individual membrane compartments with a diameter of 40 μm. (Figure 2A,B). The same trend was also evident within smaller membrane compartments of 20 μm (Figure 2C). Notably, the majority of the liquid protein condensates displayed a peripheral localization pattern after a few minutes. This peripheral localization can be attributed to the larger contact area between liquid protein condensates and lipid membranes at the periphery of the microcompartments (Figure 2B) and underscores the impact of membrane topography as a potent biophysical parameter capable of regulating the localization patterns of liquid protein condensates.

**Figure 3:**
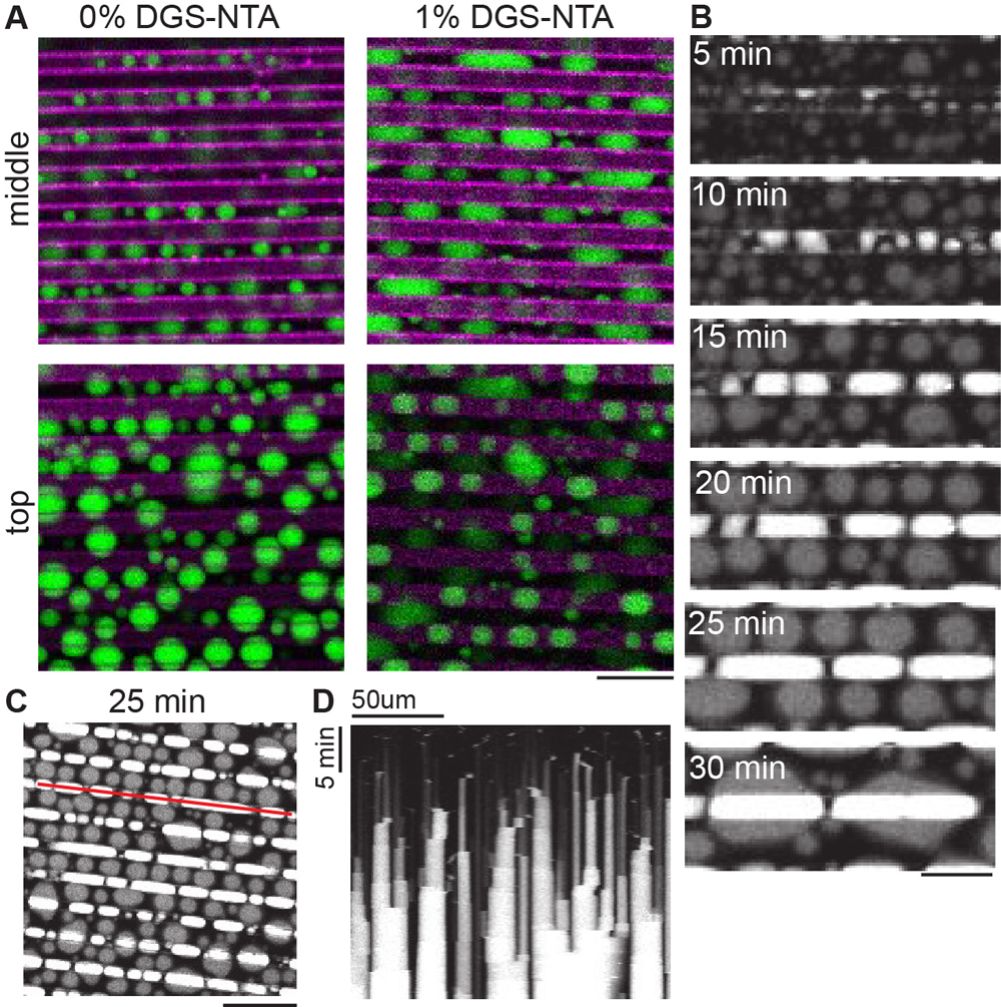
Membrane grooves mediate the stretching of liquid protein condensates along the length axis of membrane grooves. (A) Liquid protein condensates (green) were incubated for 15 min on grooved membrane surfaces (magenta) with 0% and 1% DGS-NTA, respectively. Confocal images were acquired on the upper membrane level (top) and 2.5 μm lower (middle). The grooves have a width and depth of 5 μm. The distance between grooves is 5 μm. Scale bar: 20 μm. (B) Over time, liquid protein condensates outside the grooves and inside the grooves fuse into larger liquid protein condensates. The membrane contact area of lipid condensates outside the grooves remains approximately spherical (gray), the lipid condensates within the grooves (white) are stretched (time points: 15 min to 25 min). Scale bar: 20 μm. (C) A kymograph was generated at the location indicated by the red line. Scale bar 50 μm. (D) Kymograph illustrating the fusion of liquid protein condensates along a membrane groove over time. (B, C) The grooves are 5 μm wide and 7 μm deep. The distance between grooves is 15 μm.

Next, to dissect the influence of groove-like membrane topographies, we engineered membrane-clad microgrooves as a topographical feature. We compared the shape of condensates within these membrane grooves relative to condensates residing on flat membrane regions adjacent to the grooves. Time-lapse confocal microscopy enabled us to image the evolution of liquid protein condensates as they grew over time due to fusion events (Figure 3A–C).

At the initiation of our experiment, when the droplets were small compared to the width of the grooves, we observed a random distribution of the condensates (Figure 3C). However, as the condensates progressively enlarged over time, we noted the cladding of membrane grooves, accompanied by the stretching of liquid protein condensates along the length axis of the grooves. The stretching of condensates was observed on membranes with 2% DGS, but not on membranes without DGS (Figure 3A), demonstrating that the interaction of liquid condensates with lipid membranes plays an important role for the observed shape remodeling of liquid condensates. Our observation can be attributed to capillary effects, revealing a potential mechanism for the shape adaptability and responsiveness of liquid protein condensates to preformed membrane grooves. Intriguingly, this shape adaptability along membrane edges also raises the possibility that liquid protein condensates could serve as nonspherical templates upon which forces may act to push on cellular membrane grooves, for example in the context of lamellipodial membrane protrusions and cell migration.

## Condensate Assembly on Membrane Topographies Smaller than the Liquid Protein Condensates

Thus far, we have primarily characterized the distribution of liquid protein condensates within membrane microcavities, and the dimensions of the membrane compartments were larger or comparable to the dimensions of the condensates themselves. However, in living cells, lipid membranes also undergo dynamic topographical changes on the nanoscale. Consequently, liquid protein condensates within cells may encounter topographies smaller than their own dimensions, prompting a fundamental question: How do topographies smaller than liquid protein condensates affect their spatial distribution?

**Figure 4:**
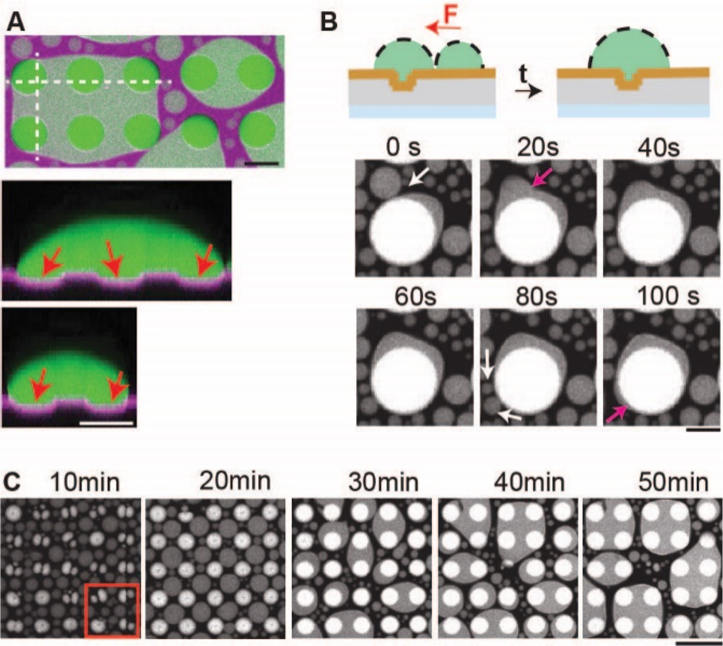
Lipid membrane surfaces with topographical structures modulate larger scale liquid protein condensates. (A) Liquid protein condensate (green) on top of a microstructured lipid membrane (magenta) and corresponding side views. Diameter of microcavity: 20 μm. Red arrows point toward microcavities. Scale bar: 20 μm. (B) Directed fusion of liquid protein condensates toward a liquid condensate, that adheres to a microcavity. White arrows: condensate before fusion event. Red arrow: Condensate after fusion event. Diameter of microcompartment: 40 μm. Scale bar: 20 μm. (C) Time-lapse confocal images of liquid protein condensates on topographically structured membranes. Red box represents data in Figure 2C. Diameter of microcompartment: 20 μm. Depth of microstructures: 7 μm. Scale bar: 50 μm.

To address this question, we determined whether the distribution of large condensates is affected by small membrane topographies. To do this, we imaged liquid protein condensates that had grown larger than the underlying membrane microstructures. Interestingly, we observed a distinctive phenomenon in which microtopographies function as pins, that shape the contours of liquid protein condensates, which extended across two or more adjacent compartments (Figure 4A,C). These observations not only underscore the importance of small-scale membrane topographies on the distribution and morphology of liquid protein condensates in the context of model membranes but also provide more general insights into the mechanisms by which protein architectures interact and respond to membrane features in complex cellular environments.

In addition to the pinning effect described above, we also observed the phenomena of a directed motion of liquid protein condensates, attributed to the capillary effect within a system of proximal liquid condensates and microcompartments (Figure 4B). Forces and capillary effects have previously been postulated as regulatory factors governing the behavior of liquid protein condensates. With our assay, we were equipped to experimentally address these phenomena in a controlled way.

Cellular processes are governed by fundamental physical mechanisms, and a current scientific frontier is unraveling how nanoscale cellular building blocks interact to form mesoscale structures and understanding the regulation of these structures. In this study, we engineered a cell-free assay to study the interaction of liquid protein condensates with topographically structured membranes by using purified proteins and membrane-clad PDMS microstructures. This cell-free approach allowed us to study the role of membrane topography in controlling the spatial distribution and shape of liquid protein condensates. The microstructures can be generated in arrays providing opportunities for the experiment parallelization.

While biomolecular mechanisms in living cells are often entangled within complex interaction networks, this cell-free approach enables the deconstruction of mechanisms for liquid protein condensate spreading on lipid membranes. However, acknowledging the inherent limitations of minimal model systems is crucial, as they focus on specific parameters and lack the complexity of a cell. In our minimal model system, we reconstituted condensates and engineered microstructures of tens of micrometers. This prompts the question about scalability to the smaller dimensions characterizing cellular condensates and cellular membrane protrusions. The 3D arrangements of cellular condensates, featuring dimensions often ranging from hundreds of nanometers to a few micrometers, are challenging to investigate on membrane topographies using standard confocal microscopy. Despite potential nonlinear scaling due to different volume-to-surface ratios and surface tension, our experiments provide an experimental demonstration that surface topography influences condensate localization and offers insights into the trends of how the localization is affected. In addition, our model system serves as powerful visualization assay, enabling the characterization of liquid protein condensate effects on topographically structured membranes.

When the proteins PRM_4_ and SH3_4_ were mixed and added to the membranes, liquid condensates assembled. These condensates attached to the membrane component DGS-NTA through the histidine-tag of PRM_4_. We studied liquid condensate formation on membranes with user-defined topography of grooves and circular microstructures. Using circular microstructures we found that liquid protein compensates preferentially accumulated at the rim of these microstructures. As this geometry resembles the intracellular, bottom contour of adherend cells our data suggest that membrane edges may represent effective cues for accumulating or enriching membrane interacting liquid protein condensates. Similarly, membrane grooves mimicking semienclosed membrane topographies showed stretched liquid protein condensates along the membrane grooves. As this geometry resembles the intracellular geometry of lamellar membrane protrusions, our findings suggest that such membrane geometries may represent effective regulators to mold liquid protein condensates along the protruding cell edges. Our results may have further interesting consequences, e.g., for the generation of feedback loops for membrane remodeling processes, because liquid protein condensates can recruit molecular force generators^9^ or modulate membranes themselves.^26,33^

Considering the complexity of living cells, the localization of liquid protein condensates may be determined by additional competing or complementary parameters. Moreover, in living cells the time and length scales of condensate formation may be specific to condensate composition and other cellular parameters. In other cell-free experiments it has for instance been shown that biophysical properties of the lipid membranes are regulators for condensate size and assembly. The formation of liquid protein condensates occurs above a concentration threshold and consequently, the recruitment of proteins to lipid membranes can serve as a mechanism for local protein enrichment and subsequent condensate formation at lipid membranes.^17^ Further, the reduced mobility of membrane-bound proteins, in contrast to freely diffusing proteins, contributes to the stabilization of relatively small protein condensates.^17^

In our experimental system, we employed protein concentrations above the threshold for condensate formation in solution. This approach enabled us to investigate the impact of an additional biophysical membrane parameter—namely, membrane topography. The resulting formation of large condensates was visualized in 3D on membrane-clad topographies generated by photolithography. Our results display localization patterns of liquid protein condensates in dependence on membrane topography and thus untangle, on a systems level, membrane topography as a general regulator for condensate assembly.

In summary, our work represents an approach to study liquid protein condensates on custom-engineered nonuniform membrane topographies. Further, we demonstrated the modulation of condensate shape and distribution through capillary forces mediated by membrane topographies and membrane wetting. Our work thereby bridges concepts of macroscopic capillary effects with the microscale world of cellular membranes, giving us valuable insights into how molecules are organized within cells by membrane topography—a fundamental biophysical parameter. We envision that future miniaturization of membrane topographies, coupled with super resolution imaging and controlled experimental conditions fostering condensate size regulation, hold large promise for unveiling the quantitative dependencies of condensate regulation by membrane topography.

## Supporting Information Available

Additional experimental details, materials, and methods including fabrication of microstructured membrane supports, supported lipid membranes, protein purification, microscopy and software (PDF)
