# Supplementary material for: Lipid Membrane Topographies Are Regulators for the Spatial Distribution of Liquid Protein Condensates
Source: Nano Lett. 2024 Apr 5;24(15):4330–5. doi: 10.1021/acs.nanolett.3c04169 (PMC11036382; doi:10.1021/acs.nanolett.3c04169)
Supplement: Supplementary file 1 — nl3c04169_si_001.pdf [file nl3c04169_si_001.pdf]

Lipid membrane topographies are regulators for the spatial distribution of liquid protein condensates

*Chae Yeon Kang, Yoohyun Chang, Katja Zieske**

Biophysics, Max Planck Institute for the Science of Light, 91058 Erlangen, Germany

Additional experimental details, materials, and methods

Fabrication of microstructured membrane supports

The two-dimensional geometry of the microstructures was determined by the patterns on a chrome mask. Microstructured surfaces were engineered by photolithography and soft molding [1]. First, photoresist (mr-DWL 5, micro resist technology) structures of about 7 μm height on top of Si wafers were generated by photolithography and the wafers were subsequently coated with trimethylchlorosilane (Sigma-Aldrich). Then, degassed PDMS (Sylgard184, monomer to cross-linker ratio 10:1, Dow Corning) was poured on top of the Si wafer and glass coverslides where pressed manually into the liquid PDMS, leaving a thin PDMS layer between the Si wafer and the glass coverslides. Finally, the PDMS was baked overnight at 60°C and the glass coverslides with the microstructured PDMS carefully separated from the wafer. These PDMS-clad glass coverslides were used as support for generating supported lipid membranes. Before generating lipid membranes, the PDMS surface was treated for 1 min with oxygen plasma.

Supported lipid membranes

Supported lipid membranes were generated by using a vesicle fusion approach. To generate small unilamellar vesicles for vesicle fusion, DOPC, DGS-NTA.NI (Avanti Polar Lipids) and

0.05% DiI (Fast DiI, ThermoFisher) were added to a glass vial and dried under an N_2-stream. The glass vial was placed into a desiccator for 30 min, followed by resuspending the lipids at a concentration of 4mg/ml in membrane buffer (25mM Tris, pH 7.5, 150mM KCl) for 30min at 37°C. The lipid solution was sonicated to generate small unilamellar vesicles, aliquoted and frozen at -20°C until further use.

To generate supported lipid membranes, small unilamellar vesicles were further diluted to a concentration of 0.5mg/ml, added to the sample chamber, supplemented with 4mM $CaCl_2$ and incubated for 30 min at 37°C. After washing the membrane with membrane buffer, the buffer was replaced with KMEI buffer (10mM Imidazole, pH 7.5, 150mM KCl, 1mM $MgCl_2$, 1mM EGTA).

Protein purification and labeling

Purification protocols were adapted from previously published protocols [2]. In short $SH3_4$ containing a N terminal GST tag, PRM_4 (with a cysteine containing tag for labeling) and PRM_4-Histidine tag were expressed in E. coli strain BL21(DE3). Bacteria were cultured to OD600 ~0.7 at 37°C, induced with 1mM IPTG and harvested after overnight incubation at 20°C. The bacteria were lysed by sonication.
PRM_4 protein constructs had an N terminal Tev-cleavable MBP tag. PRM_4 proteins were purified by Ni-NTA affinity chromatography, cleaved with TEV protease and further purified by ion exchange chromatography (Capto HiRes S column, Cytiva), followed by a Superdex 200 column. The Superdex 200 column was equilibrated in KMEI buffer (10mM imidazole, pH 7.5, 150 mM KCl, 1mM EGTA, 1 mM $MgCl_2$).
$SH3_4$ was purified with glutathione-agarose affinity chromatography, cleaved with TEV protease, further purified by ion exchange chromatography (Capto HiRes Q column, Cytiva) and dialyzed overnight into KMEI buffer.
Proteins were concentrated, frozen in liquid nitrogen and stored at -75°C until further use.
PRM_4 (with cystein for labeling) was labeled with AF488 (Jena Bioscience).

The plasmids pMAL-Abl-PRM 4R (Addgene plasmid # 112087) and pGEX SH3(2)-4R (Addgene plasmid # 112090) were gifts from Michael Rosen [2]. We thank Yoohyun Chang for help with generating PRM_4 (with a cysteine containing tag for labeling) and PRM_4-

Histidine tag. TEVSV (for purification of TEV protease) was a gift from Helena Berglund (Addgene plasmid # 125194).

Microscopy

Images were acquired using a laser scanning fluorescence microscope (LSM 980, Zeiss) equipped with a 20x objective (Plan-Apochromat 20x/ 0.8, ∞/0.17, Zeiss). All assay were performed in KMEI buffer and at room temperature.

Software

ImageJ/Fiji was used for Image analysis.

Chat GPT-3.5 was used to optimize the language of the text.

References

[1] K. Zieske and P. Schwille, "Reconstitution of self-organizing protein gradients as spatial cues in cell-free systems," *Elife*, vol. 3, p. e03949, 2014, doi: 10.7554/eLife.03949.

[2] P. Li *et al.*, "Phase transitions in the assembly of multivalent signalling proteins," *Nature*, vol. 483, no. 7389, pp. 336–340, 2012, doi: 10.1038/nature10879.
